# Identification of a novel 15‐gene expression signature predicting overall survival of human colorectal cancer

**DOI:** 10.1002/ctm2.258

**Published:** 2020-12-24

**Authors:** Chengfei Jiang, Yue Zhao, Binbin Yuan, Hang Chang, Bo Hang, Antoine M. Snijders, Jian‐Hua Mao, Xiaoping Zou, Pin Wang

**Affiliations:** ^1^ Department of Gastroenterology, Nanjing Drum Tower Hospital The Affiliated Hospital of Nanjing University Medical School Nanjing China; ^2^ Department of Gynecology The First Affiliated Hospital of Nanjing Medical University Nanjing China; ^3^ Biological Systems and Engineering Division Lawrence Berkeley National Laboratory Berkeley California

Dear Editor,

In this study, we developed a robust and clinically applicable 15‐gene prognostic signature for colorectal cancer (CRC) patients using our multi‐step bioinformatics analysis strategy. So far, no multigene expression signature is available for CRC in China where the tumor incidence is rapidly increasing.

Globally, this disease is the fourth most common of all cancer types and third leading cause of cancer mortality, with 1.8 million incident cases in 2017.^1^ Currently, the 5‐year relative survival is 65% for CRC patients, but much lower (12%) for stage IV. With the application of next‐generation sequencing and microarray technologies, several genomic biomarkers have emerged to stratify CRC patients and predict clinical outcome. Of these, the RT‐PCR‐based 7‐gene *Oncotype* DX colon cancer panel^2^, ^3^, ^4^ has been utilized as a clinical tool for the prediction of recurrence risk for stage II and III CRCs. However, there is still an urgent need to develop better and more robust gene signatures in this area. In this study, we developed a clinically applicable and robust prognostic signature for CRC patients using our multistep bioinformatics analysis strategy.^5^, ^6^, ^7^, ^8^

For our gene expression signature development, we first identified 738 genes (971 gene probe IDs) that were consistently deregulated in CRC versus normal colon tissue in six transcriptome datasets (Figure 1A and B, Figure S1A, Table S1). Kaplan‐Meier survival analysis together with Cox regression was used to evaluate whether their expression levels were associated with overall survival (OS) in GSE17536 dataset. Out of 738 genes, expression levels of 78 genes were associated with OS (*P* < .05) (Figure 1C, Table S2). These genes were significantly enriched for GO biological processes related to wound healing, collagen fibril organization, endothelial cell proliferation, and apoptosis (Figure S1C; *P* < .05).

**FIGURE 1:**
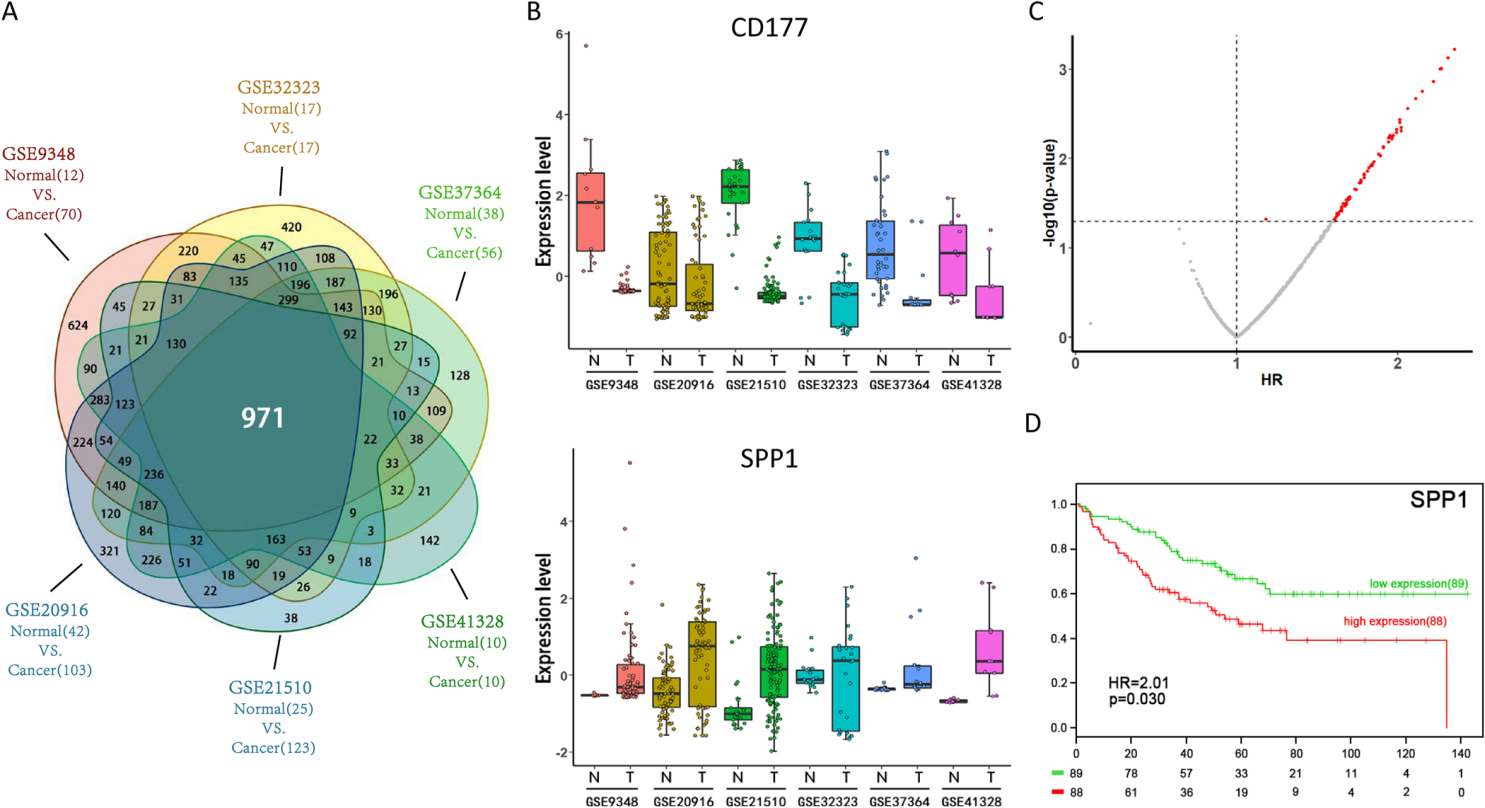
Identification of genes consistently deregulated in human colorectal cancers (CRCs) and significantly associated with overall survival (OS) of CRC patients. (A) Venn diagram of genes significantly and consistently deregulated (more than fivefold change and adjusted *P*‐value < .05) in CRC compared to normal colon tissue gene expression across six publicly available gene transcript datasets. (B) Two representative genes significantly and consistently up‐ or downregulated in CRC (T) compared to normal colon (N) tissue gene expression across all six datasets. (C) Volcano plot of the hazard ratio (HR) and *P*‐value of the association of 738 genes with OS. The 78 genes that were significantly associated with the OS of CRC (HR, *P* < .05) are highlighted in red. (D) Kaplan‐Meier survival analysis for the representative gene SPP1 from the 78 genes significantly associated with OS in CRC patients. The CRC patient cohort was divided into two groups based on the median value. The log‐rank *P*‐value and HR of the curve comparison between the high expression (red) and low expression (green) groups is shown

We then employed a cross‐validation method to resample the 373 patients in TCGA‐COAD (where TCGA is the cancer genome atlas) into 100 randomly selected training (248 cases) and test sets (125 cases) (Figure 2A). We utilized training sets to define a signature for prognosis, and to build a scoring system and prediction model, whereas test sets were used for validation. Multivariate Cox regression was carried out on all 100 training sets to identify which of the 78 genes were significantly associated with OS. Genes were ranked based on the frequency of selection in the Cox models. We next employed concordance statistics for Cox modeling to further refine the gene set with respect to their goodness‐of‐fit in survival models. Specifically, step‐wised inclusion of candidate genes into the Cox regression model based on their rank order indicated a saturated concordance statistic using the top 15 genes (Figure 2B). A 15‐gene prognostic score for a CRC patient was defined as the linear combination of logarithmically transformed gene expression levels weighted by average Cox regression co‐efficient obtained from 100 training sets (Table S3). Based on the average cut point score from the training sets, patients were divided into three prognostic groups. The OS rates of good, intermediate, and poor groups for all samples in TCGA‐COAD were significantly different based on Kaplan‐Meier analysis and log‐rank test (*P* = .0001; Figure 2D). In each test set, we compared the hazard ratios (HRs) of the intermediate versus good and poor versus good outcomes (Figure 2C). The distribution of the three survival outcome groups varied significantly across stage (*P* = .0061; Figure S2A), with increased numbers of patients in the “poor” group with increasing stage. Nevertheless, our signature significantly separated the good from the poor outcome groups for stage II, III, and IV CRC patients (*P* < .05; Figure S2B‐E). Kaplan‐Meier survival analysis in the TCGA‐COAD dataset stratified by subtype annotations (chromosomal instability [CIN], genome stable [GS], microsatellite instability [MSI], and hypermutated‐single nucleotide variant [HM‐SNV])^9^ did not show an enhancement in a particular subtype (Figure S3). Taken together, these findings demonstrate that our 15‐gene signature has clear discriminative capability to stratify CRC patients based on good versus poor prognosis.

**FIGURE 2:**
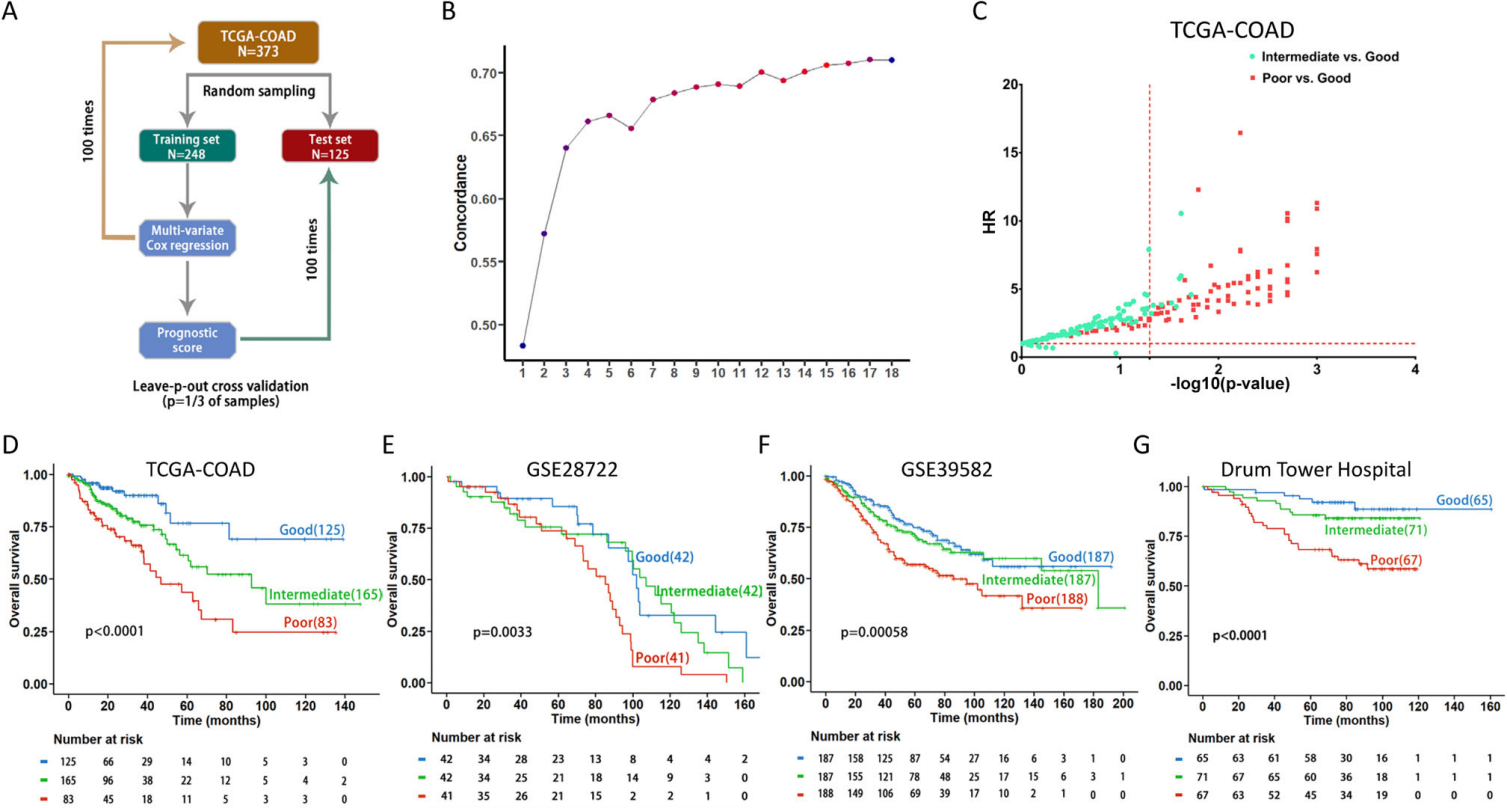
Development and validation of a multigene prognostic signature and scoring system for CRC. (A) Workflow used to generate a prognostic score system for CRC using a resampling/cross‐validation approach with Cox regression analysis. (B) Concordance statistic: Step‐wise inclusion of candidate genes into the Cox regression model. (C) HR analysis in the TCGA‐COAD dataset; (D) Kaplan‐Meier survival analysis for the TCGA‐COAD test cohort using the 15‐gene expression‐based prognostic scores. The log‐rank *P*‐value of the curve comparison between the groups is shown. (E and F) Independent validation of the 15‐gene signature in two CRC datasets GSE28722 and GSE39582, respectively. The log‐rank *P*‐value of the curve comparison between the groups is shown. (G) Independent validation of the 15‐gene prognostic score system using 203 CRC patient samples from the Nanjing Drum Tower Hospital. The patients were divided into three groups based on the cutoff of prognostic score calculated from the 135 patient training cohort. The log‐rank *P*‐value of the curve comparison between the groups is shown

To independently validate this signature, we performed Cox regression analysis in GSE28722 and GSE39582 to calculate the Cox regression co‐efficient for each of 15 genes, using the same method described above. As shown in Figure 2E and F, high prognostic score patients had a significantly shortened OS compared to low score patients (GSE28722: *P* = .0033; GSE39582: *P* = .00058). Moreover, the signature and the prognostic prediction model were tested in a cohort of 203 patients with stage I or II CRC from Nanjing Drum Tower Hospital (Figure S4). Gene expression in formalin‐fixed paraffin‐embedded (FFPE) specimens was measured using an mRNA hybridization‐based assay.^10^ OS analysis demonstrated significantly different OS rates (*P* < .0001 by log‐rank test) among the three prognostic score groups in the cohort (Figure 2G), with distribution of 32.0%, 35.0%, and 33.0% in good, intermediate, or poor prognostic score groups, respectively. These results strongly support the prognostic capability of the 15‐gene signature and score system in an independent Chinese patient cohort with early‐stage CRC.

To examine whether the prognostic effects of our signature is independent of clinicopathological factors potentially associated with patient outcomes, a multivariate Cox regression analysis was carried out on all available parameters and our signature in both TCGA‐COAD (Table S5) and our own hospital cohort (Table 1 and Table S6). These data support that the prognostic effectiveness of the 15‐gene signature was independent of clinical parameters, including molecular subtypes (*P* < .05).

**TABLE 1:** Multivariate Cox regression analysis on Nanjing Drum Tower Hospital cohort

		95% CI		
Variables	HR	Lower limit	Upper limit	*P*‐ value
Baseline information				
**T grade**				.006
T3 vs T2	3.121	0.421	23.167	.266
T4 vs T2	15.619	1.696	143.832	.015
**WHO classification**				.077
Well differentiation vs moderate differentiation	1.019	0.355	2.921	.973
Poor differentiation vs moderate differentiation	0.381	0.042	3.426	.389
Mucinous adenocarcinoma vs moderate differentiation	2.529	0.771	8.303	.126
Group				.002
Intermediate vs good	1.802	0.627	5.178	.274
Poor vs good	4.206	1.693	10.446	.002

Finally, with Cox regression analysis, the prognostic power of our signature was compared with the 7‐gene panel in the *Oncotype* DX Colon Test using two datasets. In GSE17536, the median HR of our signature for poor versus good outcomes was 2.32‐fold higher compared to the 7‐gene signature, and in GSE28722, this fold difference was 1.58 (Figure S5) indicating that our 15‐gene score outperformed the 7‐gene signature in predicting CRC patient OS.

In conclusion, we identified a novel 15‐gene prognostic signature and developed a score system that robustly and reliably predicts patient OS. The signature was validated in two independent public datasets and in our hospital cohort in Nanjing, China. The signature is independent of clinical factors as well as molecular classifiers.

## CONFLICT OF INTEREST

All authors declare that there is no conflict of interest.

## ETHICS STATEMENT

This study was approved by the Ethics Committee of the Nanjing Drum Tower Hospital (document no: 2020‐040‐01), and written informed general consent was obtained from each patient.

## FUNDING INFORMATION

National Natural Science Foundation of China; Grant Number: 81802388 (to Pin Wang); Natural Science Foundation from the Department of Science & Technology of Jiangsu Province; Grant Number: BK20180120 (to Pin Wang).

## AUTHOR CONTRIBUTIONS

*Study concept and design*: Pin Wang, Xiaoping Zou, Jian‐Hua Mao, Antoine M. Snijders, and Bo Hang; *data acquisition*: Chengfei Jiang, Yue Zhao, Pin Wang, and Binbin Yuan; *statistical data analysis*: Jian‐Hua Mao, Pin Wang, and Chengfei Jiang; *manuscript drafting*: Pin Wang, Chengfei Jiang, Bo Hang, and Jian‐Hua Mao; *funding and study supervision*: Pin Wang and Xiaoping Zou; *manuscript editing*: all authors.

## Supporting information

Supporting Information

Supporting Information

Supporting Information

## Data Availability

Requests for the datasets utilized for the current study will be reviewed and considered by the corresponding authors.
