# Supplementary material for: Identification of a novel 15‐gene expression signature predicting overall survival of human colorectal cancer
Source: Clin Transl Med. 2020 Dec 24;10(8):e258. doi: 10.1002/ctm2.258 (PMC7759535; doi:10.1002/ctm2.258)
Supplement: Supplementary file 1 — Supporting Information [file CTM2-10-e258-s001.pdf]

LETTER TO EDIOR

Identification of a novel 15-gene expression-based prognostic signature for overall survival of human colorectal cancer

Chengfei Jiang et al

Supplementary Material

Methods

Ethics Statement: This study was approved by the Ethics Committee of the Nanjing Drum Tower Hospital (document no: 2020-040-01), and written informed general consent was obtained from each patient.

Publicly Available Patient Datasets and Screening for OS-associated Genes

In order to gather a large sample size of CRC transcriptomic data for signature development, we used the following datasets: GSE9348, GSE20916, GSE21510, GSE32323, GSE37364, and GSE41328, from the NCBI Gene Expression Omnibus, which include both CRC and normal colorectal tissue cases, and GSE17536, GSE28722, GSE39582 and TCGA-COAD datasets, which contain CRC transcriptome and related clinical information.

To identify genes that consistently deregulated in CRC, we performed a meta-analysis to identify such genes across the six public datasets using GEO2R. The criteria for significant differential expression of a gene were: 5-fold change in expression level and an adjusted $p<0.05$. Next, Kaplan-Meier survival analysis together with Cox regression was used to evaluate the impact of high or low expression levels of the consistently deregulated genes on OS in

GSE17536, and the log-rank test was used to determine statistical significance. GSE28722 and GSE39582 were used as independent validation datasets.

Gene Expression-Based Signature and Prognostic Risk Score Development

To develop a multigene prognostic signature, we first generated the training sets by performing 100 random selections of 373 patients from TCGA-COAD. The remaining patients after each selection were used as test sets. A forward-conditional Cox regression with the 78 genes significantly associated with OS in GSE17536 was carried out using SPSS (IBM, version 24) to further select genes independently associated with OS in each training set. The frequency by which each gene was selected during the forward-conditional Cox regression analysis in the 100 training sets was calculated. Genes were ranked based on their frequency and we used a concordance statistic to determine the optimal number of genes in the prognostic signature [1].

We then repeated Cox regression on all 100 training sets using the 15-gene signature as covariates and the forced entry (enter) method to acquire the coefficient for each gene. For each gene, the 100 coefficient values were averaged to estimate the true coefficient of each gene. The prognostic score of each patient was calculated based on the expression levels of the 15 genes and average Cox regression coefficients (formula see below).

$$Prognostic\ score = \sum_{i=1}^{15} (Cox\ regression\ coefficient\ for\ gene\ i\ ) * (gene\ i\ expression\ level\ )$$

All patients in the training sets were divided into three groups based on prognostic score. The tertiles of prognostic score in each training set were identified as cut points and averaged across 100 training sets to obtain the optimal value of cut points. Based on these cut-points, each patient was assigned into one of three groups: “good”,” intermediate” and “poor” prognostic

outcome. Kaplan-Meier analysis and log-rank test were conducted among the test sets to verify the coincidence of the predictive power of a gene signature, as described in our previous studies [2-5].

To determine the biological functions enriched in the 78-gene set associated with OS, we performed Gene Ontology enrichment analysis using the ClueGO plug-in in Cytoscape (version 3.7.1). ClueGO was run using default parameters and $p<0.05$ as a cut-off for significance [6].

Validation of the 15-Gene Score System Using Two Public Datasets

125 samples from GSE28722 dataset and 562 samples from GSE39582 were utilized as two independent validation sets. We analyzed the mRNA expression levels and OS status for the 15-gene signature using the same Cox regression method described above, which gave a set of new coefficients for the 15-gene signature. Prognostic scores of each sample in the two validation sets were calculated and split into tertiles: "good", "intermediate" and "poor" prognostic outcome. The OS status from the three groups was analyzed by Kaplan-Meier analysis and differences in survival among the groups was tested using the log-rank test.

The 15-Gene Score is Independent of Clinicopathological Features of CRC

To confirm the independence of the 15-gene signature in its prognostic function, we analyzed available clinicopathological factors using multivariate Cox regression analysis on their HR values, in relation to the 15-gene score.

We also analyzed the distribution of prognostic groups based on the 15-gene signature in the four CRC molecular subtypes. Kaplan-Meier analysis of OS within the four subtypes was

performed and the survival curves were compared using the log-rank test. HRs with 95% CI were calculated and the percent distribution of the patients in three prognostic scores for each of the subtypes was recorded.

Comparison with the Oncotype DX Colon Cancer 7-Gene Signature

Using the same methodology described above using patient cohorts from GSE17536 (177 patients) and GSE28722 (125 patients), the prognostic ability of our 15-gene signature was compared with a previously developed Oncotype DX 7-gene signature [7-9]. Briefly, we performed a multivariate Cox regression analysis of the 7 genes, with 100 training sets for GSE17536 (118 patients) and GSE28722 (83 patients) separately. Coefficients for each of the 7 genes were averaged and prognostic scores for all the patients were then calculated as described above. Based on the scores, we divided the patients in 100 test sets into tertiles (good, intermediate, and poor), with the cut point scores being recorded and averaged. All the patients for each cohort were then separated into three groups based on these cut points and Kaplan-Meier analysis of OS was then used to compare the prognostic performance of the 7-gene and 15-gene signatures. HR values were calculated for each testing set for the "poor" group in comparison to the "good" group.

Validation of the 15-Gene Expression Signature Using an Independent Hospital Cohort

A total of 203 patients with stage I and II CRC were enrolled in this study. All of the patients were pathologically diagnosed and underwent colorectal radical surgery between 2007 to 2015 at Nanjing Drum Tower Hospital, Nanjing University. Patients were followed up until July 2020 for survival status. The inclusion criteria were as follows: (1) Stage I and II patients diagnosed

with CRC for at least 5 years; (3) complete patient clinical and survival information; (4) recent clinical follow-up by this study; and (5) FFPE sample taken at the first-time pathological diagnosis.

The expression measurement of the 15 prognostic genes and 5 reference genes (*ACTB, RPLP0, GUSB, TFRC, GAPDH*) in the FFPE samples were performed using a mRNA hybridization assay previously described [5]. It should be noted that, in the clinical validation study, we used a different mRNA detection technology platform as compared to the public datasets used. The latter consisted of CRC transcriptome data obtained using microarray or RNA-seq.

We performed Cox regression on 100 resampling training sets consisted of 135 patients and the coefficient values for each gene were averaged as weights for calculating prognostic score. All samples in the training sets were sorted and equally trisected based on the prognostic scores. The tertiles of prognostic score in each training set were identified as cut points. Then, we averaged the upper and lower tertiles separately to obtain the optimal value of cut points. Patients with prognostic score were divided into three groups: "good"," intermediate" and "poor". Kapan-Meier analysis with log-rank test and HR with 95% CI were conducted among the test sets with remaining 68 patients to verify the coincidence of the prognostic power of the gene signature. Multiple clinical and pathological factors including age at diagnosis, gender, TNM stage, WHO classification, primary tumor site in relation to the 15 genes-based prognostic groups were used as parameters for univariate as well as multivariate Cox regression analyses.

Statistical Analysis

Statistical methods used in this work are described in different sections above.

References

1. Harrell FE Jr, Lee KL, Mark DB (1996) Multivariable prognostic models: issues in developing models, evaluating assumptions and adequacy, and measuring and reducing errors. Stat Med 15:361–387
2. Wang P, Wang Y, Hang B, et al (2016) A novel gene expression-based prognostic scoring system to predict survival in gastric cancer. Oncotarget 7:55343–55351
3. Chen E-G, Wang P, Lou H, et al (2018) A robust gene expression-based prognostic risk score predicts overall survival of lung adenocarcinoma patients. Oncotarget 9:6862–6871
4. Zhao Y, Yang S-M, Jin Y-L, et al (2019) A Robust Gene Expression Prognostic Signature for Overall Survival in High-Grade Serous Ovarian Cancer. J Oncol 2019:3614207
5. Zhu L, Wang H, Jiang C, et al (2020) Clinically applicable 53-Gene prognostic assay predicts chemotherapy benefit in gastric cancer: A multicenter study. EBioMedicine 61:103023
6. Bindea G, Mlecnik B, Hackl H, et al (2009) ClueGO: a Cytoscape plug-in to decipher functionally grouped gene ontology and pathway annotation networks. Bioinformatics 25:1091–1093
7. Clark-Langone KM, Sangli C, Krishnakumar J, Watson D (2010) Translating tumor biology into personalized treatment planning: analytical performance characteristics of the Oncotype DX Colon Cancer Assay. BMC Cancer 10:691
8. Srivastava G, Renfro LA, Behrens RJ, et al (2014) Prospective multicenter study of the impact of oncotype DX colon cancer assay results on treatment recommendations in stage II colon cancer patients. Oncologist 19:492–497
9. Brenner B, Geva R, Rothney M, et al (2016) Impact of the 12-Gene Colon Cancer Assay on Clinical Decision Making for Adjuvant Therapy in Stage II Colon Cancer Patients. Value Health 19:82–87

Supplementary Figures

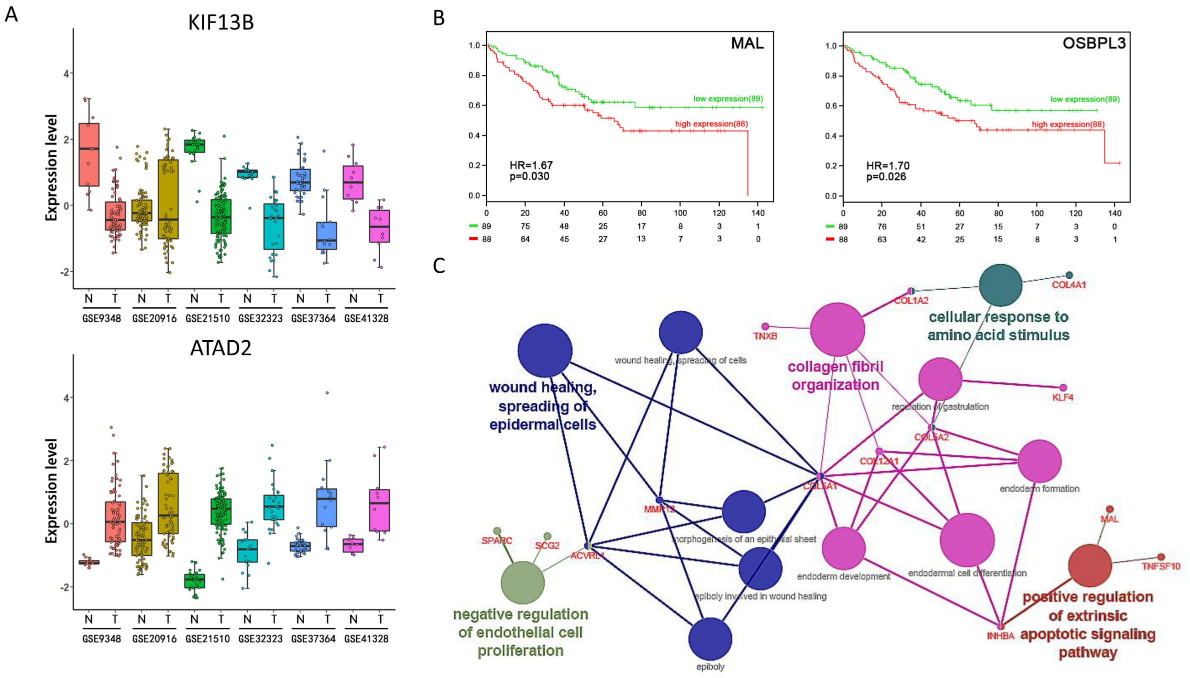

Figure S1. Bioinformatics analyses using TCGA-COAD dataset. **A**. Two representative genes significantly and consistently de-regulated in CRC (T) versus normal colon (N) tissue gene expression across all six data sets. **B.** Kaplan-Meier survival analysis for two representative genes from 78 genes significantly associated with OS in CRC patients. **C.** Gene ontology (GO) enrichment analysis of 78 genes significantly associated with OS in CRC patients. Significantly enriched GO biological processes are shown as individual nodes ($p<0.05$). Functionally related terms have the same color and the node size represents the significance of the term enrichment. Genes associated with a functional term and listed in red.

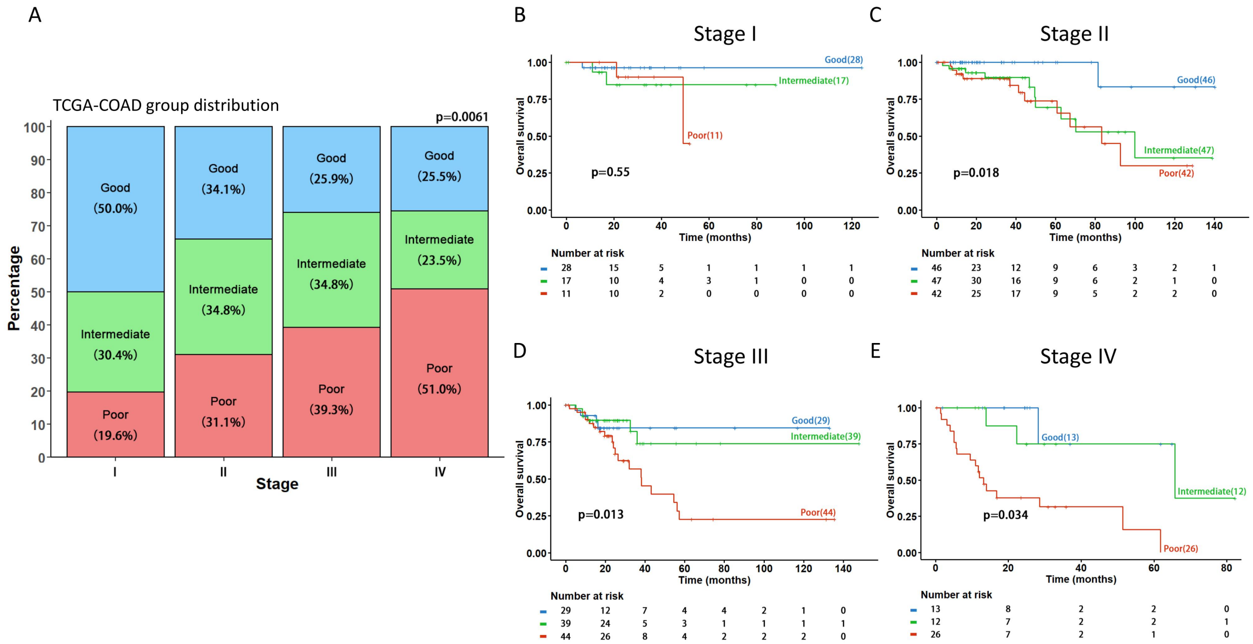

Figure S2. A. Percentage distribution of three prognostic score groups at different stages in the TCGA-COAD dataset. The p-value was obtained by Chi-Square test. **B-E.** Kaplan-Meier curves with log-rank p-values for the three groups at stage I to IV, respectively.

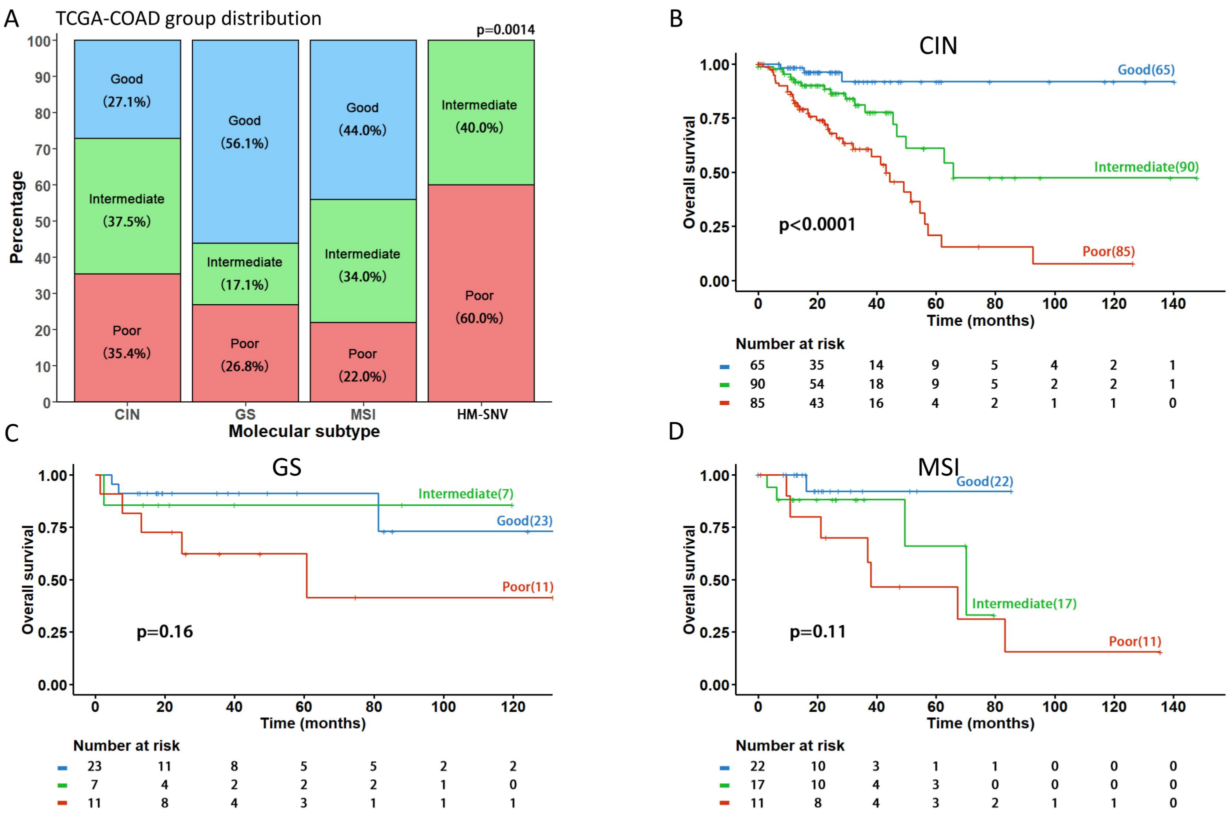

Figure S3. A. Percentage distribution of three prognostic score groups in four molecular subtypes of CRC, i.e., CIN (chromosomal instability), GS (genome stable), MSI (microsatellite instability), and HM-SNV (hypermutated-single nucleotide variant). **B.** Kaplan-Meier curves for the three groups in the above subtypes, respectively. The HM-SNV subtype was only represented by 10 patients, preventing us from assessing the accuracy of our scoring system in this subtype.

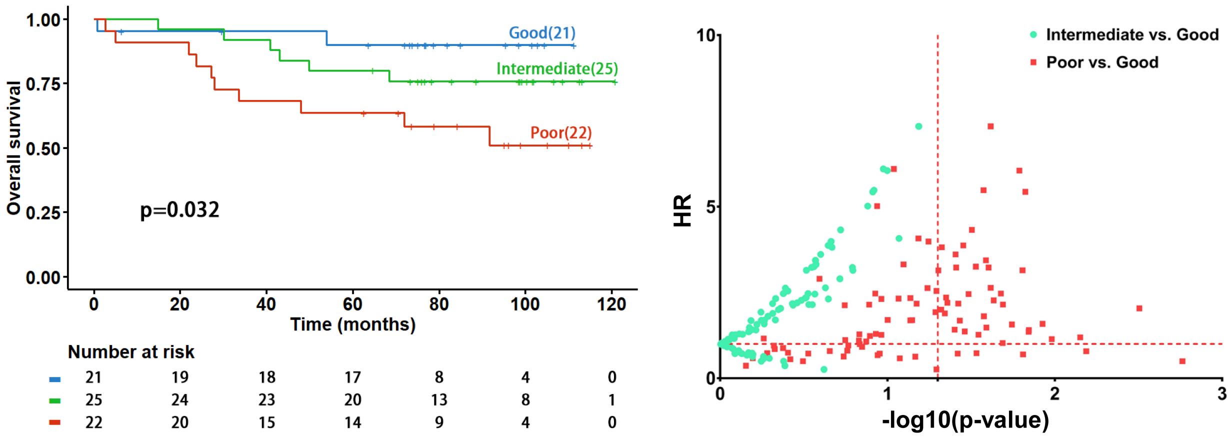

Figure S4. A. A representative K-M plot from the test set containing 68 patients from the Drum Tower Hospital cohort. **B**. HR distribution of intermediate vs. good and poor vs. good groups. *** indicates P < 0.001.

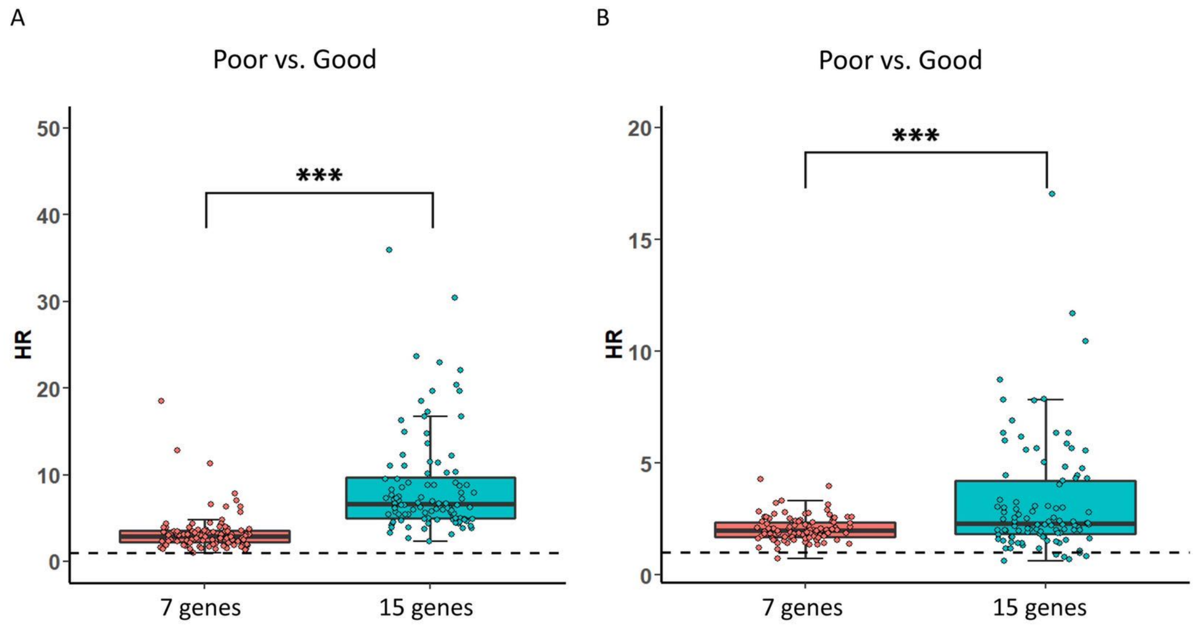

Figure S5. Comparison of the HR obtained from 100 test sets between the 7-gene panel and our 15-gene signature in GSE17536 (**A**) and GSE28722 (**B**). For each of these datasets, the HR values calculated for poor vs. good were plotted. *** indicates P < 0.001 based on Wicoxon test.
