# Supplementary material for: Identification of a novel 15‐gene expression signature predicting overall survival of human colorectal cancer
Source: Clin Transl Med. 2020 Dec 24;10(8):e258. doi: 10.1002/ctm2.258 (PMC7759535; doi:10.1002/ctm2.258)
Supplement: Supplementary file 3 — Supporting Information [file CTM2-10-e258-s003.pdf]

Supplementary Tables

Table S2. 78 genes significantly associated with the overall survival (OS) of patients with CRC (*p*-value < 0.05) (highlighted in yellow)

Gene symbol	HR	P - value
ADAMTS12	2.35	0.0005988
EMP1	2.31	0.0007376
COL11A1	2.27	0.0009786
FUCA1	2.26	0.0009909
INHBA	2.22	0.00137
HOMER1	2.15	0.001756
TTLL6	2.11	0.002108
CDKN2B	2.06	0.002737
SPP1	2.01	0.003645
PLCD1	2.01	0.003911
COL5A2	2.02	0.004465
TMEM37	1.99	0.004694
COL1A2	2.02	0.004905
COL5A1	1.99	0.005135
NAT2	1.97	0.00547
TMPRSS2	1.95	0.005533
LOC101928195///LOC100996643///MTHFD1L	1.96	0.005663
APOBR	1.95	0.005691
SPARC	1.96	0.005794
NDRG2	1.94	0.005858
TTC22	1.96	0.005957
SPON1	1.94	0.006493
FAM189A1	1.91	0.007395
COL10A1	1.91	0.007525
ADAM12	1.88	0.008976
COL12A1	1.89	0.009227
ACOT11	1.85	0.01085
ACVRL1	1.84	0.01149
ACP1	1.84	0.0119
CD177	1.83	0.012
PID1	1.82	0.0126
MYC	1.81	0.01381
SCG2	1.79	0.01485
SLC39A5	1.79	0.01491
KDELC1	1.79	0.01516
MIR22///MIR22HG	1.79	0.01628
UGGT2	1.77	0.01731
KLRC4-KLRK1///KLRK1	1.77	0.01782
COL4A1	1.77	0.01845
ATAD2	1.76	0.01869
GPA33	1.73	0.02194
KLF4	1.74	0.02207
MZT1	1.7	0.0266

CTHRC1	1.7	0.0271
FGD4	1.69	0.02745
OSBPL3	1.7	0.02792
TNFSF10	1.69	0.02805
BCL2L15	1.69	0.0281
ACOX1	1.69	0.02882
MLXIP	1.68	0.02898
QPCT	1.68	0.03102
PLA2G16	1.66	0.03175
SCARA5	1.68	0.03191
MAL	1.67	0.03205
TTC38	1.67	0.03262
LGALS2	1.68	0.03272
KLK15	1.67	0.03281
IMPA2	1.67	0.03314
SLC44A4	1.67	0.03387
CAPN9	1.65	0.03475
FAP	1.65	0.03612
MMP12	1.64	0.03836
KIRREL	1.64	0.03858
SNORD77///SNORD76///SNORD74///GAS5///SNORD44///SNORD47///SNORD80///SNORD79///SNORD81	1.65	0.03864
NFE2L3	1.63	0.04036
ACO2	1.63	0.04062
EIF5A2	1.62	0.04113
SLC35D1	1.63	0.04188
STC2	1.62	0.04235
SCAMP2	1.62	0.04295
C10orf99	1.62	0.04343
HRCT1	1.62	0.04349
TNXB//TNXA	1.62	0.04367
EPB41L4B	1.61	0.04605
SEMA4G	1.18	0.04733
CORO2A	1.6	0.04834
KIF13B	1.61	0.04885
NAAA	1.6	0.04905
ASNS	1.6	0.0493
WDR78	1.6	0.04987
SHISA2	1.59	0.05013
SELENBP1	1.59	0.05047
LEF1	1.6	0.05102
HSD11B2	1.58	0.05407
LRRC19	1.58	0.05531
EFHC2	1.57	0.05699
SLC9A2	1.57	0.05734
PIGR	1.57	0.05774
TMEM72	1.57	0.05873
PXMP2	1.57	0.05989
CLMN	1.57	0.06011
MORC4	1.57	0.06076
FKBP1B	0.64	0.06129
TFCP2L1	1.56	0.06195

EDNRA	1.56	0.06342
VIPR1	1.55	0.06362
HSD17B2	1.56	0.06387
ZNF575	1.55	0.06412
TMEM30B	1.54	0.06563
TPSG1	1.55	0.06568
ETHE1	1.54	0.0686
DPF3	1.54	0.06992
CCNYL1	1.54	0.06995
TDGF1P3///TDGF1	1.54	0.07067
COL1A1	1.54	0.07213
ACADS	1.53	0.07232
ABCC13	1.53	0.07257
LOC389332	1.53	0.07397
NXPE4	1.52	0.07451
PITPNM3	1.53	0.07558
EPHX2	1.53	0.07693
DUSP14	1.52	0.07724
RAD54B	1.52	0.0783
CHGB	1.52	0.07921
GPSM2	1.51	0.08352
IL6R	1.52	0.08354
GCG	1.51	0.08366
TSPAN7	1.51	0.08495
GPD1L	1.5	0.08624
UCA1	1.5	0.08635
SMPD1	1.5	0.08657
STYK1	1.5	0.08851
RETSAT	0.67	0.08869
LOC100996506///RETSAT	0.67	0.08869
ARRDC4	1.49	0.0894
PDCD2L	1.49	0.08995
SLC25A25-AS1	1.49	0.09192
RAE1	1.49	0.09198
APPL2	1.49	0.0922
ACACB	1.48	0.09665
TMEM100	1.48	0.09841
SLC51B	1.48	0.09882
HIST1H1C	1.48	0.09997
GRHL1	1.47	0.1004
TCN1	1.47	0.1026
VWA5A	1.47	0.1041
SLC22A3	1.47	0.1044
ANLN	1.47	0.1049
CXCL11	1.47	0.1051
C4orf19	1.47	0.1063
NEU4	1.46	0.1082
IGH	1.46	0.1084
GSTA1	1.46	0.1085
SAMD9	1.46	0.1094
RCN1	1.46	0.1103
MYO1A	1.46	0.1109
SLC25A34	1.46	0.1121

MAOA	1.46	0.1132
CAPN13	1.45	0.1162
SCNN1B	1.45	0.117
LARGE2	1.45	0.1178
ENTPD5	1.45	0.1182
LIPH	1.44	0.1213
PARM1	1.44	0.1216
ECH1	1.44	0.1225
TAF1A	1.44	0.1226
KIAA0513	1.44	0.1255
FBLIM1	1.44	0.1258
MAOB	1.43	0.1289
ANGPTL1	1.43	0.1302
BTNL3	1.42	0.1373
DENND2A	1.42	0.1388
PLAU	1.42	0.14
WISP1	1.42	0.1421
RCAN2	0.71	0.143
FLNB	1.41	0.1466
SRI	1.41	0.1478
ZAK	1.41	0.1491
MT1X	1.41	0.1493
MPND	1.4	0.1555
STAP2	1.4	0.1564
PPARD	1.4	0.1574
TMPRSS3	1.39	0.1577
DHDDS	1.4	0.1607
B3GNT6	1.4	0.1608
UGT2A3	1.4	0.1611
C6orf136	1.39	0.1618
AKR1B10	1.39	0.1623
JAM2	1.39	0.1624
LOC727916	1.39	0.1635
SORBS2	1.39	0.1669
RHBDL2	1.39	0.1682
WNT5A	1.39	0.1691
KRT6B	1.38	0.1705
CDK1	1.38	0.1735
FAM3D	1.38	0.1737
GOLM1	1.38	0.174
PYY	0.72	0.1767
MOB3B	1.38	0.1774
LOC100652777///PLA2G10	1.38	0.1775
CP	0.73	0.179
ADH1C	1.37	0.1791
CHP2	1.37	0.1842
AGT	1.37	0.1855
DISP2	1.37	0.1858
GALNT6	1.36	0.1885
GRAMD3	1.36	0.1893
TRPM6	1.36	0.1918
FAM132A	1.36	0.1919
EPB41L3	1.36	0.1928

MIA-RAB4B///RAB4B	1.36	0.1949
NEBL	1.36	0.1953
PDE9A	1.36	0.1962
C15orf38-AP3S2///AP3S2	1.36	0.1963
ISX	1.36	0.1993
CNNM4	1.36	0.1993
LRRC8E	1.35	0.2005
GPAT3	1.35	0.203
MUC12	1.35	0.2034
SLC22A23	1.35	0.2048
WDR75	1.35	0.2064
FTH1	1.35	0.2066
UGP2	1.35	0.2075
ANGPT2	1.35	0.2098
NKD1	1.34	0.2118
SLC6A6	1.34	0.2124
BICD1	1.34	0.2133
AKR7A3	1.34	0.2137
DGAT2	1.34	0.2137
MMP1	1.34	0.2151
CD36	1.34	0.2177
KIAA1161	1.33	0.2194
SLC13A2	1.34	0.2206
IGHM	1.33	0.2216
UGDH	1.33	0.2217
PLCL2	0.75	0.2222
SLC9A3R1	1.34	0.2224
HECTD3	1.33	0.2227
BMP2	1.34	0.2248
ASCL2	1.33	0.2248
SPIB	1.33	0.225
SGK2	1.33	0.2254
C7	1.33	0.2265
CMBL	1.33	0.2266
NEK2	1.33	0.2279
SLC9A1	1.33	0.2303
CDH3	1.33	0.2303
RIPK2	1.33	0.2312
CDKL1	1.33	0.2323
ANK2	1.33	0.2338
TCEA3	1.33	0.2344
EXOSC7///CLEC3B	1.32	0.2355
GDPD3	1.32	0.2366
ITPKA	1.32	0.2371
MAMDC2	1.32	0.238
SEMA6D	1.32	0.241
OSBPL7	1.31	0.2447
CEL	1.32	0.2448
RCL1	0.76	0.2475
CLU	1.31	0.2477
PKIB	1.31	0.2488
MALL	1.31	0.2523
GLTP	0.76	0.253

HJURP	1.31	0.2553
SORD	1.31	0.2555
UBD///GABBR1	1.31	0.2557
SCIN	1.31	0.2565
TMEM54	1.31	0.2577
DSCC1	1.31	0.2591
FGFBP1	1.31	0.2595
CES2	1.3	0.266
CCL15-CCL14///CCL15	1.3	0.2662
TPH1	0.77	0.2664
CCDC68	1.3	0.2672
CA1	0.77	0.2678
CPNE8	1.3	0.2703
ACSS2	1.3	0.2705
FGFR2	1.3	0.2709
PNO1	1.3	0.2719
IDH3A	1.3	0.272
PSAT1	1.29	0.2743
LZTS3	1.29	0.2766
MT2A	1.29	0.2767
TEX30	1.29	0.2773
ADH1B	1.29	0.2795
EYA2	1.29	0.2815
ANPEP	0.78	0.2858
CA4	1.29	0.2863
BEST2	1.28	0.287
KIF1C	1.29	0.2875
RPS6KA1	1.29	0.2878
XDH	1.29	0.2896
LAMA1	1.28	0.2898
BEST4	1.28	0.2901
HHLA2	0.78	0.2943
DSC2	1.28	0.2946
NUF2	1.28	0.295
PROX1	1.28	0.2961
PPP2R3A	1.28	0.302
BCHE	1.27	0.3022
C1orf210	1.28	0.3025
ANKRD9	1.27	0.3044
PLCG2	1.27	0.3057
CHST5	1.27	0.3063
CD44	1.27	0.3068
GZMB	1.27	0.3133
TRAF5	0.79	0.3145
LIFR	1.27	0.3165
SLC22A18	1.26	0.3173
SLC26A2	1.26	0.3175
TIMP3	1.27	0.3194
C2CD4A	1.26	0.3211
DUXAP10	1.26	0.3219
B3GNT7	0.79	0.322
IFITM1	1.26	0.3221
NHSL1	1.27	0.323

AXIN2	1.26	0.3254
ARHGAP44	1.26	0.3262
LRRFIP2	1.26	0.3265
CITED2	1.26	0.3283
TUBAL3	1.26	0.3317
PPID	1.26	0.3334
NR5A2	1.25	0.334
CEACAM1	1.25	0.3359
KPNA2	1.25	0.3383
SNX24	1.25	0.3384
HPGD	1.25	0.3389
MUC2	1.25	0.3412
TRPM4	1.25	0.3423
CIDEC	1.25	0.3428
ACAA2	1.25	0.3457
BLOC1S1-RDH5///RDH5	1.25	0.3469
NAP1L1	1.25	0.3487
CHGA	1.25	0.3488
FHL1	1.24	0.3537
CXCL2	1.24	0.3565
MXI1	1.24	0.3567
EPHX4	0.8	0.3576
MT1E	0.8	0.3584
ABCA8	1.24	0.359
BACE2	1.24	0.3602
TMEM246	1.24	0.3635
FOXQ1	0.81	0.3671
PMAIP1	1.24	0.3673
MT1H	1.24	0.3701
PEX26	1.24	0.3716
KLK1	1.23	0.3721
CXCL12	1.24	0.3723
TST	1.23	0.3723
CIDEB	1.23	0.3745
CA12	1.23	0.3749
EGFL6	1.23	0.375
KRT20	0.81	0.3752
MYOT	1.23	0.3753
CFD	1.23	0.3779
MBOAT1	1.23	0.3781
CDHR5	1.23	0.3792
UGT1A3///UGT1A1///UGT1A4///UGT1A9///UGT1A5///UGT1A6///UGT1A7///UGT1A8///UGT1A10	1.23	0.3807
UGT1A3///UGT1A1///UGT1A9///UGT1A5///UGT1A8	1.23	0.3807
UBE2T	1.23	0.3832
SNTB1	1.23	0.3836
ARNTL2	1.23	0.3851
PPM1H	1.23	0.3862
LRMP	1.22	0.3935
ANXA9	1.22	0.3941
HMGCS2	1.22	0.3943

CHI3L1	1.22	0.3946
CADPS	1.22	0.395
GBA2	0.82	0.3964
KIAA0895	1.22	0.3969
SLC17A4	0.82	0.3995
SULT1B1	1.22	0.4005
ENDOD1	1.22	0.402
KCNIP4	1.22	0.4046
CRNDE	0.82	0.4107
MGAT4A	1.22	0.411
LRRC66	1.21	0.4122
CPM	1.21	0.4128
CKB	1.21	0.413
FAM83E	1.21	0.4158
CA7	1.21	0.4173
CHP1	1.21	0.4197
SCN7A	0.83	0.4223
MGLL	1.21	0.4238
KRT23	1.21	0.4238
ARID3A	0.83	0.4258
ARRB1	0.83	0.4262
DPT	1.21	0.4289
SIM2	0.83	0.4291
ABHD3	1.2	0.4337
FAM47E-STBD1///FAM47E///STBD1	1.2	0.437
DEFB1	1.2	0.4382
FZD5	1.2	0.4398
LRP8	1.2	0.4402
CDK4	1.2	0.4406
TRIP13	1.2	0.4413
SIAE	0.83	0.4414
CPNE5	1.2	0.4418
GBA3	1.2	0.4447
CHRDL1	1.2	0.446
ADTRP	1.2	0.4463
VILL	1.2	0.4466
HOXD1	0.84	0.4474
TOP1MT	1.19	0.4496
VDR	1.19	0.4498
UBE2S	0.84	0.4533
EPB41L4A	0.84	0.4567
FXYD3	1.19	0.4601
TCFL5	1.19	0.4614
SLC36A1	1.19	0.462
ZBTB7C	1.19	0.4655
HRASLS2	1.19	0.4657
ST6GALNAC1	1.19	0.4662
E2F6	1.19	0.4662
RILP	1.19	0.4665
ZFAS1	0.84	0.4705
SLX1B-SULT1A4///SLX1A-SULT1A3///SULT1A4///SULT1A3	1.18	0.4723
PTPRH	1.18	0.4728

PMEPA1	1.19	0.4729
POLR1D	1.18	0.4749
ADAMTS2	1.18	0.4787
TGIF1	1.18	0.4807
MET	1.18	0.4836
PAG1	1.18	0.4848
CKS2	0.85	0.4854
DIO2	1.18	0.4886
C11orf86	0.85	0.4901
SCGN	1.18	0.4901
VSIG2	1.18	0.4923
DHRS1	1.18	0.4947
CFB	0.85	0.4972
CCL23	1.17	0.5002
KLB	1.17	0.5006
ECHDC2	1.17	0.5037
FCGBP	1.17	0.5041
KIF16B	1.17	0.5063
GUCA2B	1.17	0.5081
RIPK3	1.17	0.5085
ENPP3	1.17	0.5094
C6orf223	1.17	0.5102
CYP4F12	1.17	0.5106
TINCR	1.17	0.5119
GUCA2A	0.86	0.5145
DDX21	1.17	0.51575
C12orf29	1.17	0.5173
OGN	1.17	0.5178
P2RX4	0.86	0.521
PADI2	1.16	0.5213
SULT1A2	0.86	0.5234
CXCL1	0.86	0.5246
ACAA1	1.16	0.5261
IFITM3	1.16	0.5278
SMPDL3A	1.16	0.5281
TRANK1	0.86	0.5303
CMSS1	1.16	0.5327
SQRDL	1.16	0.5339
PIGZ	1.16	0.534
CNNM2	1.16	0.5356
ABCC3	1.16	0.5373
BRINP3	1.16	0.5377
PHLPP2	1.16	0.5377
SHROOM4	1.16	0.5386
TSPAN1	1.16	0.542
B3GALT5	0.87	0.5422
TPX2	1.15	0.5423
P2RY1	1.15	0.5429
MMP3	1.15	0.5479
PAQR5	1.15	0.5484
TEX11	1.15	0.549
MEIS3P1	1.15	0.5491
USP2	1.15	0.5559

MOGAT2	1.15	0.5595
FOXP2	1.15	0.5597
SLC30A10	0.87	0.5602
GCNT3	1.15	0.5612
DPEP1	0.87	0.5625
CD3EAP	1.14	0.5665
ATP2A3	1.14	0.5688
PTGDR	1.14	0.5715
CLIC5	0.88	0.5715
IL1R2	0.88	0.5724
CTPS1	0.88	0.5729
CFI	1.14	0.5738
MIER3	1.14	0.5749
GREM2	0.88	0.5758
AJUBA	1.14	0.5776
GART	1.14	0.5785
GNA11	1.14	0.5799
FAM107A	1.14	0.5808
CELSR3	1.14	0.5846
TMEM220	1.14	0.5867
SULT1A1	0.88	0.5876
SLC6A8	1.13	0.5955
SIPA1L2	0.88	0.5961
SST	1.13	0.6
PHF19	0.88	0.6017
NR3C2	1.13	0.6032
ENC1	1.13	0.6033
PGM1	1.13	0.6075
UGT1A1///UGT1A4///UGT1A9///UGT1A6///UGT1A8///UGT1A10	1.13	0.6078
PUS7	1.13	0.6094
SLC1A1	1.13	0.61
RELL1	0.89	0.6104
SCNN1G	1.13	0.6105
ABCG2	1.13	0.6123
ASPA	1.13	0.6126
PCK1	1.13	0.6162
GTF2IRD1	1.12	0.6172
DACH1	1.13	0.618
CSGALNACT1	0.89	0.619
CYP2C18	1.12	0.6192
LIMA1	1.12	0.6195
WT1	0.89	0.6214
ANO5	0.89	0.6229
ASAP3	1.12	0.6287
HEPACAM2	0.89	0.6317
LARP6	1.12	0.6333
PINK1	0.9	0.6378
AQP8	0.9	0.6404
CLDN1	1.11	0.6457
FAM92A1	1.11	0.6482
NXPE1	1.11	0.6509
IQGAP2	1.11	0.651

SOX9	1.11	0.6517
CEACAM7	1.11	0.6531
NR1H4	1.11	0.6532
PROCR	0.9	0.6549
CLDN2	1.11	0.6552
CES3	1.11	0.6556
NUFIP1	1.11	0.6581
PSMA7	1.11	0.659
PRKCB	1.11	0.6598
RUNDC3B	0.9	0.6616
CCL28	0.9	0.6618
CXCL6	0.9	0.664
MT1F	1.11	0.6647
MSX2	0.9	0.6696
TMEM56	0.9	0.6697
LSM8	0.91	0.6737
FKBP1A-SDCBP2///SDCBP2	1.1	0.6763
CLCA4	0.91	0.6786
APCDD1	1.1	0.6833
CDH19	1.1	0.6866
SLC25A20	1.1	0.687
PTGER4	1.1	0.6876
SMIM14	1.1	0.6894
CD1D	0.91	0.6896
PAPSS2	0.91	0.6899
SRPX2	1.09	0.6992
TMEM206	1.09	0.7027
CASP7	0.091	0.7033
NME1	0.91	0.7042
HELLS	0.92	0.7081
CNTN3	1.09	0.7087
CWH43	1.09	0.7096
LDHD	0.92	0.7097
BBIP1	1.09	0.7108
KLHL29	1.09	0.7119
PDK4	0.92	0.712
TLCD2	1.09	0.7142
LPCAT4	1.09	0.7212
PRR5L	0.92	0.7232
UNC5C	0.92	0.7297
REG3A	1.08	0.7313
TMEM61	0.92	0.7324
NKX2-3	1.08	0.7344
TNFSF15	1.08	0.7434
UNC5CL	0.93	0.7439
SHROOM3	0.93	0.7464
ABHD5	0.93	0.7473
C1orf115	0.93	0.7482
HILPDA	0.93	0.7489
WIPF2	0.93	0.75
MYO15B	1.08	0.7501
PDE3A	1.08	0.7505
EPHB1	0.93	0.7506

AZGP1	1.08	0.7515
MACC1	1.08	0.7526
PLEKHA7	1.07	0.7585
EFNA5	1.07	0.7606
GDF15	0.93	0.7617
A1CF	1.07	0.762
SLC16A4	1.07	0.7654
ITM2C	1.07	0.7665
DHRS11	0.93	0.7674
MSX1	0.93	0.7693
CXCL8	1.07	0.7724
UTP4	1.07	0.7726
CDHR2	1.07	0.7729
STMN2	0.93	0.7742
AHCYL2	1.07	0.7748
SLC41A2	1.07	0.7752
AHNAK	1.07	0.7767
SPINK5	1.07	0.7782
NAP1L2	1.07	0.7783
ATP11A	0.94	0.7836
MUC4	1.07	0.7838
SLC26A3	0.94	0.7843
EXOSC3	0.94	0.7849
RNF125	1.07	0.7867
ZNF503	1.07	0.7869
SEMA6A	1.06	0.7897
LOC143286	0.94	0.7901
PRKAR2B	1.06	0.792
FXYD1	1.06	0.7938
MS4A12	1.06	0.7957
ECT2	0.94	0.7961
PRKACB	1.06	0.8037
BCAR3	1.06	0.8075
SLCO4A1	0.94	0.8095
RFC3	1.06	0.8119
PLCD3	0.95	0.8125
PLAC8	1.06	0.8137
MEP1B	1.06	0.8178
LDLRAD3	0.95	0.8183
ZZEF1	1.06	0.8189
SLC16A9	1.05	0.8198
PCBP1-AS1	1.05	0.8206
LPAR1	1.05	0.8224
ITM2A	0.95	0.8258
ANO10	1.05	0.827
CA2	1.05	0.8274
MYO1C	1.05	0.8278
FABP6	1.05	0.8291
ECI2	1.05	0.8294
WNT2	1.05	0.8337
RNF43	1.05	0.8351
TMEM171	0.95	0.8352
SLC2A13	0.95	0.8357

SIDT1	1.05	0.8363
CAPN5	1.05	0.8373
HIGD1A	1.05	0.8392
SLC12A2	1.05	0.8394
CDK6	0.95	0.8411
CALM3///CALM2///CALM1	1.05	0.8464
CXCL3	1.05	0.8501
CEBPB	1.05	0.8506
TSPAN5	0.96	0.8512
ETFDH	0.96	0.8519
AKR1C1	0.96	0.8551
BCAS1	1.04	0.8574
PLP1	1.04	0.8589
KBTBD11	1.04	0.8631
RAPGEFL1	1.04	0.864
CPT2	0.96	0.8642
CYP4X1	0.96	0.8676
S100A9	0.96	0.8677
ELOVL6	1.04	0.8695
MXD1	1.04	0.8701
CDC42SE2	0.96	0.8791
TJP3	0.97	0.8803
NEDD4L	0.97	0.8812
GPT	1.04	0.8823
ABI3BP	0.97	0.8869
RAB27A	1.03	0.89
CGN	1.03	0.8944
CKMT1A///CKMT1B	0.97	0.8952
MMP7	1.03	0.8974
SLC25A23	1.03	0.905
MMP28	1.03	0.9053
SFRP1	1.03	0.9054
FAM63A	1.03	0.9056
MIR4680///PDCD4	1.03	0.9077
TCF21	0.97	0.9078
AOC1	1.03	0.9098
SLC22A18AS	0.98	0.916
SPPL2A	1.02	0.9176
ATP5S	1.02	0.9183
C2orf72	0.98	0.9188
VSNL1	1.02	0.9224
AMN	0.98	0.9259
SGK1	1.02	0.9262
FLVCR2	0.98	0.9264
ST6GALNAC6	1.02	0.9284
ABCE1	0.98	0.9286
POU2AF1	1.02	0.9299
ACSL6	0.98	0.9304
PTP4A1	0.98	0.932
CLDN23	1.02	0.9321
LOC339166///WSCD1	1.02	0.9333
FAM107B	0.98	0.9388
SI	0.98	0.9408

KIAA1211	0.98	0.9424
PRSS12	0.98	0.9436
GOT1	0.98	0.9438
C2orf40	0.98	0.9444
AHCY	1.02	0.945
METTL7A	0.98	0.9452
FMO5	0.99	0.9508
TRIB3	0.99	0.9516
EDN3	0.99	0.9526
TP53I3	1.01	0.9529
C16orf62	1.01	0.9544
THRB	0.99	0.9561
PHLDA1	1.01	0.9591
PDE7B	0.99	0.9597
SLC39A10	1.01	0.9622
ARL14	1.01	0.9633
MT1G	0.99	0.9724
MT1M	0.99	0.9727
C2orf88	1.01	0.9743
DIMT1	0.99	0.9751
TGFBI	0.99	0.9765
FRMD3	1	0.9839
TP53INP2	1	0.9844
DHRS9	1	0.9851
GCNT2	1	0.987
PLCE1	1	0.9882
GALNT12	1	0.9911
IGSF9	1	0.9924
SLC4A4	1	0.993
UBE2C	1	0.993
EIF4E3	1	0.995
TMCC3	1	0.9978
GGT6	0.99	0.99818
TACSTD2	1	0.9984
TOX	1	0.9998

Table S3. The 15 gene names, accession numbers and canonical discriminant function coefficients

Gene	Accession No.	Coefficient
TTC22	NM_017904	-0.3122
ACOT11	NM_015547	-0.3018
WDR78	NM_024763	-0.3017
NAT2	NM_000015	-0.2847
KIF13B	NM_015254	-0.2652
IMPA2	NM_014214	-0.1933
ATAD2	NM_014109	-0.1824
CD177	NM_020406	0.0253
SPP1	NM_000582	0.0853
SCG2	NM_003469	0.1303
OSBPL3	NM_145320	0.145
PLA2G16	NM_007069	0.1914
MAL	NM_002371	0.2244
TMPRSS2	NM_005656	0.3193
C10ORF99	NM_207373	0.3791

Table S4. The functions of 15 genes in the prognostic signature

Gene	Gene name	Gene function
MAL	Myelin and lymphocyte protein	Encodes a membrane protein belonging to the MAL family of proteolipids. The protein has been localized to the endoplasmic reticulum of T-cells
KIF13B	Kinesin family member 13B	Encodes a kinesin-like motor protein, which may be involved in reorganiazing the cortical cytoskeleton. It may regulate angiogenesis
OSBPL3	Oxysterol-binding protein-related protein 3	Encodes a member of the oxysterol-binding protein (OSBP) family. The protein is involved in the regulation of cell adhesion and organization of the actin cytoskeleton.
SCG2	Secretogranin II	Encodes a neuroendocrine secretory granule protein that is the precursor for biologically active peptides. It regulates apoptosis and angiogenesis.
TMPRSS2	Transmembrane Serine Protease 2	Encodes an androgen-regulated transmembrane serine protease. Serine proteases are involved in numerous physiological and pathological processes.
CD177	CD177 antigen, NB1	Encodes a glycosyl-phosphatidylinositol (GPI)-linked cell surface glycoprotein that plays a role in neutrophil activation. CD177 is considered to represent neutrophils
ACOT11	Acyl-coenzyme A thioesterase 11	Encodes a member of the acyl-CoA thioesterase family which catalyze the conversion of activated fatty acids to the corresponding non-esterified fatty acid and coenzyme A.
IMPA2	Inositol monophosphatase 2	Encodes an inositol monophosphatase that catalyzes the dephosphorylation of inositol monophosphate and plays an important role in phosphatidylinositol signaling.
SPP1	Secreted phosphoprotein 1 (Also osteopontin)	Encodes secreted phosphoprotein 1, also named osteopontin (OPN), a key protein in extracellular matrix by function in the attachment of osteoclasts to the mineralized bone matrix.
NAT2	N-acetyltransferase 2	Encodes an enzyme that functions to both activate and deactivate arylamine and hydrazine drugs and carcinogens. Polymorphisms in this gene are also associated with higher incidences of cancer, including colorectal cancer (CRC) (da Silva 2011).
PLA2G16	Phospholipase A2 group VI	Phospholipase A2 is highly expressed in adipose tissue, with function in breaking down phospholipids. Specifically, the A2 phospholipase regulates the levels of phosphatidylcholine.

C10ORF99	chromosome 10 open reading frame 99	a novel human antimicrobial peptide with largely unknown cellular function. It has the highest expression level in normal colon tissue. It is down-regulated in colon cancer tissues and cell lines.
ATAD2	ATPase family AAA domain containing protein 2	The protein contains two AAA (ATPases Associated with diverse cellular Activities) domains, which belongs to AAA family that often perform chaperone-like functions
TTC22	Tetratricopeptide Repeat Domain 22	Encodes a protein with seven tetratricopeptide (TPR) repeats which are important for functions associated with the cell cycle, transcription, protein transport complexes and protein-protein interactions.
WDR78	WD repeat domain 78	Encodes a member of the WD-repeat protein family of unknown function. It is highly expressed in motile cilia-containing tissues.

Table S5. Multivariate Cox regression analysis in TCGA-COAD (319 cases)

Variables		Hazardous Ratio	95% CI		p-value
			Lower Limit	Upper Limit	
**Baseline Information**					
	**Diagnosis age**	1.032	1.010	1.054	0.004
	**Gender**	0.816	0.488	1.365	0.439
	**T Grade**				0.074
	T2 vs. T1	0.644	0.069	6.038	0.700
	T3 vs. T1	0.654	0.041	10.335	0.763
	T4 vs. T1	1.627	0.099	26.740	0.733
	**N Grade**				0.325
	N1 vs. N0	0.728	0.196	2.703	0.635
	N2 vs. N0	1.187	0.332	4.252	0.792
	**M Grade**				0.012
	M0 vs. M1	2.773	1.413	5.443	0.003
	Mx vs. M1	1.455	0.690	3.069	0.324
	**Stage**				0.645
	Stage II vs. Stage I	1.130	0.108	11.811	0.919
	Stage III&IV vs. Stage I	2.117	0.172	26.080	0.558
	**Pathological classification**	1.679	0.796	3.541	0.173
	**Primary Tumor Site**	0.746	0.386	1.442	0.384
**Molecular Subtype**					
	**Fraction genome alteration**	87.266	5.677	1341.493	0.001
	**Aneuploid score**	0.933	0.877	0.992	0.026
	**Mutation count**	1.000	0.999	1.001	0.701
	**Molecular subtype**				0.588
	GS vs. CIN	0.636	0.257	1.576	0.329
	MSI vs. CIN	1.493	0.391	5.709	0.558
	POLE vs. CIN	5.648	0.060	531.533	0.455
**Group**					0.001
	Intermediate vs. Good	2.638	1.038	6.704	0.042
	Poor vs. Good	5.100	2.135	12.185	0.000

Table S6. Univariate Cox regression analysis on data from Nanjing Drum Tower Hospital

Variables	HR	95% CI		p-value
		Lower limit	Upper limit	
**Baseline Information**				
**Diagnosis age**	1.016	0.991	1.042	0.223
**Gender**	1.144	0.617	2.121	0.668
**T Grade**				0.000
T3 vs. T2	4.715	0.646	34.391	0.126
T4 vs. T2	32.220	3.572	290.616	0.002
**Stage II vs. Stage I**	4.815	0.662	35.023	0.121
**WHO classification**				0.023
Well differentiation vs. Moderate differentiation	0.275	0.037	2.027	0.205
Poor differentiation vs. Moderate differentiation	2.735	1.283	5.832	0.009
Mucinous adenocarcinoma vs. Moderate differentiation	0.875	0.306	2.501	0.803
**Primary Tumor Site**	0.786	0.394	1.569	0.495
**Ki67**	0.634	1.135	2.964	0.562
**Group**				0.001
Intermediate vs. Good	1.521	0.553	4.187	0.417
Poor vs. Good	4.382	1.796	10.692	0.000
